# Supplementary material for: Are carbon emissions trading and green financial instruments synergistic? -Comprehensive quantitative research based on content analysis
Source: PLoS One. 2024 Mar 7;19(3):e0298601. doi: 10.1371/journal.pone.0298601 (PMC10919601; doi:10.1371/journal.pone.0298601)
Supplement: S1 Data — (ZIP) [file pone.0298601.s001.zip › Data/地级市Z_F工作报告.zip!/╣π╢1⁄2/╟σ╘╢╩╨/└┤╫╘╣1⁄2╓┌║┼í╛┬φ┐╦╩2╛▌═°í┐_2012.docx]

各位代表：

我代表市人民政府，向大会报告本届政府过去五年的工作，并对今后五年及今年的工作提出建议，请予审议，并请政协各位委员和其他列席人员提出意见。

一、本届政府的工作回顾

本届政府任期的五年，是我市经济社会发展取得显著成效的五年。在省委、省政府和市委的正确领导下，在市人大、市政协的监督支持下，本届政府承接上届政府开创的良好态势，紧紧依靠全市人民，积极应对国际金融危机的持续冲击，成功克服雨雪冰冻、洪涝等自然灾害带来的不利影响，全力推进经济社会的快速发展，较好地完成了市五届人大一次会议确定的各项目标任务。

（一）经济实力大幅提升。通过狠抓工业发展、积极扩大内需、大力推进“双转移”，千方百计做大经济规模。主要经济指标在连续多年高速增长、总量持续扩大的基础上，继续保持了快速增长。2011年，预计全市生产总值1286亿元，来源于清远的财政总收入221.3亿元，地方财政一般预算收入84.3亿元，分别比2006年增长1.5倍、4.98倍和3.3倍；外贸进出口总额45.5亿美元，是2006年的2倍；固定资产投资五年累计3493.7亿元，是上个五年的3倍。五年共引进项目2625个，计划总投资4323.72亿元，累计实际投入1434.65亿元。其中，引进外资项目334个，合同外资金额27.36亿美元，实际利用外资22.17亿美元。水泥、陶瓷、有色金属等传统产业继续发展壮大，电子信息、机械装备等新产业实现了初步的发展。预计2011年规模工业增加值650亿元，比2006年增长3.4倍，工业在产业结构中的比重超过60%。五年共利用园地山坡地补充耕地30万亩，新增一批规模化、标准化种养基地和国家级、省级农业龙头企业。积极实施农业“两品”（品质、品牌）工程，共获得无公害农产品认证215个、有机食品认证17个、绿色食品认证46个、国家地理标志产品12个。外向型农业加快发展，备案（注册）农产品出口基地发展到64家。预计2011年农业增加值138亿元，比2006年增长34.2%。金融机构各项存款余额超千亿元，现代化支付体系建设成效明显；交通银行等金融机构在我市新设立了分支机构，4家企业启动了上市程序。旅游业实现了新的发展，成功创建国家5A级景区1个、4A级景区9个，列全省地级市之首。预计2011年全市旅游接待人数达2602.6万人次，实现旅游收入161.9亿元，分别比2006年增长2.3倍和4.5倍。专业市场建设扎实推进，城西大道-人民路商贸物流集聚带雏形初显。预计2011年第三产业增加值403亿元，比2006年增长107.9%。

（二）城乡面貌明显改善。五年累计投入基础设施建设资金935亿元，完成了武广高铁清远段、清连高速等重大项目建设，广乐、二广、广清扩建等高速公路工程及一批公路大修工程全面推进。提前14个月完成连接建制村公路硬底化任务，基本实现镇镇通等级公路，村村通水泥路。五年共投入电网建设资金84.86亿元，是上个五年的3.31倍，顺利实现了骨干电网由110千伏向220千伏的转换。共解决116.3万农村人口的饮水安全问题，提前三年完成省下达的任务。完成堤防建设和加固303公里，病险水库除险加固工程82座，省人大议案机电排灌工程158宗。城市规划管理范围从市区扩大到全市，完成了城西大道、连江西路、沿江路、高铁站站前广场、凤城文化广场和中山公园、汝珍公园等一批市政项目新建和改建工程，建成了一批文化娱乐设施、商业综合体和住宅小区。北江水利枢纽工程成功截流，飞来湖防灾减灾工程基本完成，大燕河水利枢纽工程全面开工，湖城建设的基础工程基本完成。各县县城的中轴线建设初见成效，城市功能不断完善。投入城乡环境治理资金72亿元，城乡清洁工程、“三边”整治深入推进，宜居城乡和新农村建设试点进展顺利。广东北部生态屏障建设初见成效，“市肺”、“万村绿”行动顺利开展，全市森林覆盖率达67.8%。加强水资源和环境保护，加快淘汰落后产能，强化重点流域及重点排污企业监管，新增日污水处理能力29.5万吨，顺利完成了“十一五”节能减排目标任务。全市主要江河水质保持良好，饮用水源水质100%达标。拆解行业和陶瓷产业污染得到有效整治。成功创建成为省卫生城市、省园林城市。

（三）改革创新深入推进。重点领域改革扎实推进，完成了市县两级政府机构改革和大部门制改革试点，先后进行了两轮行政审批制度改革，初步建立起“一网式”审批和并联审批系统。金融机构改革进一步深化，市农信社统一法人工作进展顺利，城信社退出市场工作全面完成。医药卫生体制改革扎实推进，政府办基层医疗机构全部实施基本药物制度。文化体制改革获全国先进，新华书店和电影公司成功转企改制，市民族歌舞团重新组建。基层农业技术推广体系和集体林权制度改革基本完成，华侨农场改革发展取得重大进展。体制机制创新成效明显，市财政投资的工程项目全面实行代建制，建立了干部工作考核评价机制和县（市、区）目标责任考核评价体系，充分调动了各级推动经济发展的积极性。打破行政区域限制，创办了民族工业园，为“三连”地区工业发展搭建了平台。整合华侨农场国有土地资源，创办了华侨工业园。与顺德区共建了顺德清远（英德）经济合作区，创造了产业转移园合作的全新模式。科技工作开创了新局面，2011年全市专利申请量625件，专利授权量289件，分别比2006年增长2.68倍和2.01倍。产学研合作领域不断拓宽，品牌创建成效突出，共组建省级工程技术研发中心5家、省火炬计划特色产业基地3个，拥有自主创新产品13个、省名牌产品19个、中国驰名商标4件。我市被评为全国科技进步考核先进市，清城区、清新县、阳山县被评为全国科普示范县。

（四）扶贫攻坚成效显著。以“规划到户、责任到人”为重点，变“输血”式扶贫为“造血”式扶贫，扶贫开发工作取得突破性进展。产业扶贫、智力扶贫和互助金扶贫深入开展，贫困户自我发展能力明显增强。成功探索出碧桂园帮扶树山村等企业扶贫开发的新模式。全市共投入“双到”资金10.46亿元，扶持发展集体经济项目1421个，培训农村贫困劳动力20.7万人次，转移农村贫困劳动力就业4.9万人，成功帮助23.46万人脱贫，302条省级贫困村集体经济收入全部超过3万元，提前一年完成“双到”扶贫开发任务。在全省率先实施“两不具备”地区移民搬迁安置工作，预计春节前可顺利完成高寒山区196条自然村、3696户17246人的移民搬迁年度任务。完成了9405户农村低收入困难户住房改造任务，幸福安居工程示范村进展顺利。民族地区生产生活条件逐步改善，经济社会发展速度不断加快。连山、连南两个民族自治县财政一般预算收入双双突破亿元，实现了城乡免费义务教育，医疗条件和基础设施也有了较大幅度的提升。五年来，市本级财政共安排资金17.3亿元，通过专项补贴等方式转移支付给县区，有力地支持了县域经济的发展。

（五）民生事业加快发展。五年共投入民生领域资金328.61亿元，财政民生投入占一般预算支出的62.9%。五年新增城镇就业29.68万人，下岗再就业4.8万人，城镇登记失业率控制在3.5%以内，企业职工最低工资水平比2006年提高70%，共有1.28万农民工通过积分制入户城镇。预计2011年城镇居民人均可支配收入1.77万元，农村居民人均纯收入7387元，分别比2006年增长68.2%和82%。全民社保格局基本形成，社保待遇明显提高，企业退休养老待遇比2006年增长1.1倍，医保最高支付额由社平工资的2.8倍提高到3.6倍。家庭人均年收入低于1500元的困难群众全部纳入最低生活保障范围。五年新增保障性住房6463套，发放租赁补贴672户。建立了低收入群众临时价格补贴与价格上涨联动机制，共向18.46万名优抚、低保和农村五保供养对象发放价格补贴1285.8万元。学前教育三年行动计划顺利启动，普及高中教育提前一年完成，市特殊学校落成开学。省职业技术教育基地落户我市，清远高职院办学规模持续扩大，办学特色初步显现。教师工资福利待遇实现“两相当”，较好地解决了代课教师和校舍安全问题。累计创建省教育强县（区）2个、省教育强镇（街）18个、国家级示范性普通高中10所。公共文化服务体系建设加快推进，农村电影放映工作走在全省前列，农家书屋提前五年实现行政村全覆盖。市属医疗卫生资源整合初见成效，重大疾病预防控制不断加强，妇幼保健服务、农村（社区）卫生服务有新的进步。体育场所建设有序推进，农民体育健身工程全面完成。成功举办了第四届市运会，群众性体育运动广泛开展。切实发挥县镇村综治信访维稳中心作用，强化安全生产专项整治，严厉打击各类犯罪活动，安全生产和社会治安形势总体稳定。着力维护劳动者合法权益，加强企业人文关怀，和谐劳动关系得到新发展。深入开展打私、打假、打传和产品质量专项整治，大力推进食品安全工程，市场秩序不断规范。

五年来，我们扎实抓好国防动员和后备力量建设，双拥工作卓有成效，连续三届获得全省双拥模范城称号。顺利完成了第六次全国人口普查工作。人口自然增长率7.38‰，顺利完成了省下达的人口计划指标。全面完成了“五五”普法工作，法治城市、法治县（市、区）创建活动不断深入。妇女儿童和残疾人权益保护深入推进，宗教关系保持和谐，侨务、人防、气象、档案、方志、对台、红十字等工作也取得了新进展。

各位代表，过去五年，我们始终重视政府自身的改革与建设，取消和调整行政审批项目475项，政府网站建设和政务公开不断完善。应急管理体系基本建立，预防和应对突发事件能力有效提升，妥善解决了北江铊污染和血铅超标事件。公务员教育培训和管理的制度化建设进一步加强。政风建设和反腐败斗争深入开展，监察、审计等专门监督工作富有成效。积极推进民主法制建设，自觉接受人大、政协的监督和社会大众的监督，开展人大代表、政协委员约见市长活动，共办理人大代表建议346件、政协提案743件，人大代表和政协委员对办理工作的满意率不断提高。

回顾五年来的政府工作，我们深刻地体会到：必须始终把改善民生作为政府工作的出发点和落脚点，不断提高群众的幸福感和满意度；必须始终注意发挥县、镇两级政府的作用，最大限度地调动基层的积极性和创造性；必须始终发扬敢想敢试、争先进取的精神，保持全市上下奋发有为的工作状态；必须始终坚持务实实干的工作态度，注重在抓落实上下功夫；必须始终保持政府的廉洁与高效，不断提升政府自身的建设水平。上述五点，既是过去推进发展得到的宝贵启示，也是新一届政府必须秉承的基本理念。

各位代表，五年来，在清远这片土地上，我们一起经历了发展的艰辛，也共同分享了进步的喜悦。五年成绩的取得，归功于省委、省政府的正确领导，归功于历届领导班子打下的良好发展基础，归功于市委的坚强领导和科学谋划，归功于市人大、市政协与各民主党派的有效监督与大力支持，归功于全市人民、广大企业家和所有参与清远发展建设的朋友们的拼搏实干，归功于中央、省驻清各单位和驻清部队、武警官兵的鼎力支持，归功于各兄弟市、省直各单位，尤其是挂钩帮扶单位的大力帮助，也归功于港澳台同胞、海外侨胞、国际友人和社会各界的关心和参与。在此，我代表市人民政府，一并表示由衷的感谢！

在总结成绩的同时，我们也清醒地看到，我市经济社会发展还存在一些问题和隐忧：虽然我市经济总量已经跃居全省中游水平，但经济发展质量不高，资源消耗过大，环境约束趋紧，低增值产业、房地产业仍然是产业的主体，转变经济发展方式艰巨而紧迫；南北部发展差距进一步拉大，贫困面仍然相当大，特别是贫困地区的基本公共服务和基础设施水平仍然很低，实现全市区域协调发展任重道远；一些不具备开发条件的地区低效益开发和破坏性开发的情况仍然比较突出，生态环境保护的压力加大；城市化率低于全省的平均水平，中心城市功能不完善，辐射带动全市发展和服务产业发展的能力还比较弱，提高城市化水平刻不容缓；社会组织发展薄弱，优质教育、医疗、文化资源总量不足，分布不均衡，社会矛盾依然比较多，社会建设和管理的任务将更加繁重；民生保障水平还比较低，一些基本的民生问题还没有有效解决，民生事业发展与全市人民的期待仍然有较大差距。

应该看到，上述问题的存在，是一个地区在一定的发展阶段不可避免的，也是目前社会处于转型期必然会出现的。面对和解决这些问题，也正是我们今后发展的动力和潜力。当然，从政府工作来反思，也表明政府科学发展的理念、推进发展的能力、为民造福的意识还有待加强，统筹能力、行政效能、服务水平等还有待提升。特别是，一些政府工作人员得过且过、不思进取、作风散漫的情况比较突出，全力推动和服务发展的责任心和工作热情明显减退。这特别值得我们警醒。

深入剖析这些存在的问题，目的是让政府在取得巨大成绩的同时，能够保持头脑的冷静与清醒。特别是，在清远多年快速发展的情况下，更需要通过深刻的自我剖析，才能增强忧患意识，防止出现自满情绪，更好地找准工作的方向和着力点。也只有这样，才能保持全市上下奋发有为的精神状态，开启全新的征途。

二、今后五年的发展目标和工作重点

综合分析清远未来五年的发展态势，尽管面对后金融危机时期经济发展的不确定和不稳定，但全球新一轮产业布局的调整，珠三角产业转移的加快，新兴产业的迅速发展和国内市场的快速成长，都为我们提供了巨大的增长空间。特别是，随着广清城际轨道的开工建设和广乐、二广、佛清从等多条纵横贯通清远的高速公路先后建成通车，清远对接融入大广佛的条件更加具备。因此，从总体上判断，未来五年，清远仍将处于大有可为的重要战略机遇期。只要我们谋划得当，采取正确的发展战略，完全有可能在更高的层次上加快发展。

市第六次党代会敏锐地把握了区域发展的新态势，提出了全面实施桥头堡发展战略，推动清远经济社会发展新跨越的战略构想。桥头堡发展战略的提出，改变了清远在区域定位上的传统思维，是对清远城市发展、产业发展和区域发展的全新思考，理清了清远未来的发展思路，也为今后五年的政府工作指明了方向。

今后五年，政府工作的总体要求是：深入贯彻市第六次党代会精神，全面落实桥头堡发展战略，以富民强市为目标，以推动转型升级为抓手，以提升产业水平、推进城市化、统筹区域发展、增强地区软实力和持续改善民生为工作重点，加快转变经济发展方式，加大社会民生建设力度，全面统筹推进经济、社会、文化、政治、生态文明建设，为实现清远经济社会发展的新跨越奠定基础，努力把清远建设成环珠三角高端产业成长新区、全省区域协调发展示范区、大广州卫星城和华南休闲宜居名城。

按照上述总体要求，立足清远目前的实际，今后五年的政府工作，需要认真处理好三个重要的关系：

一是发展速度与发展质量的关系。单纯追求速度的经济发展模式已不再符合我市经济发展的阶段性要求，必须坚持质量优先，速度服从质量，宁可在提升发展质量的过程中暂时减速，也不能继续追求低效益、高消耗、不可持续的速度。与此同时，要通过加快转型升级，培育新一轮增长的动力，尽快形成新的产业支撑，有效应对经济下行的压力，全面实现新的加速发展。

二是经济建设与社会建设的关系。在坚持以经济建设为中心的同时，更多地注重社会建设和民生福祉。特别是，随着今后五年经济的进一步发展和城市化水平的提升，社会、民生问题将更加突出，需要政府投放更多的精力、财力和智慧，在提高社会建设、管理和服务的水平上有更大的作为。

三是产业发展与环境保护的关系。生态环境是清远最宝贵的资源，也是最大的竞争优势。绝不能以牺牲环境的代价来换取产业的发展，绝不走先污染后治理的老路。要通过推进现有产业的污染减排，淘汰落后的生产工艺，将宝贵的环境容量，留给资源消耗少、技术含量高、环境效益和经济效益好的项目。

按照市第六次党代会的总体部署，秉持上述要求和原则，提出未来五年富民强市的奋斗目标：

——到2016年，全市人均生产总值、城乡居民人均收入比2011年翻一番；第三产业增加值比重达到35%左右，高新技术产业增加值占工业增加值的比重达到10%以上，先进制造业、战略性新兴产业增加值在工业增加值中的比重明显上升；地方财政收入结构更加合理。

——到2016年，全市城镇化率达到55%以上，南部地区达到70%以上；市区绿色湖城的格局基本形成，英德、连州两个次中心城市规模扩大，功能提升；全市森林覆盖率达到70%以上，城市人均绿地面积达到18平方米以上，城镇生活污水处理率达到75%以上，城镇生活垃圾无害化处理率达到90%以上。

——到2016年，全市基本公共文化服务体系不断完善，城乡医疗保险、养老保险实现全覆盖；南北部地区城乡居民人均收入、基本公共服务水平差距缩小；城乡低收入群体的生活保障水平明显提升，人均期望寿命达到74.5岁。

未来五年的政府工作，必须致力于下述重点：

（一）发展高增值产业

经济发展的根本动力在于产业，必须紧紧抓住产业建设这个关键，强化优先发展工业的意识，加快园区基础设施配套，引进上规模的优质企业，发展培育新兴产业，提升产业发展水平。

提升传统优势产业。不再新批传统的建筑陶瓷制造和金属拆解冶炼用地，推动陶瓷、有色金属加工等传统产业改进生产工艺，加快向产业的下游和附加值高的环节延伸，提高竞争力与综合效益。大力实施农业“两品”工程，推进农产品标准化建设和管理，发展农业专业合作社，做响做强优势农产品品牌。引进有实力的大企业深度开发旅游资源，打造具有较强竞争力的旅游产品；深入挖掘优质生态、历史人文、民俗风情等资源潜力，延长旅游产业链条，做大做强旅游产业。促进房地产业健康发展。

培育新的支柱产业。整合广清城轨、职教基地和绿色湖城三大要素，打造环珠三角高端产业成长新区。充分发挥我市紧靠广佛北部产业聚集带的独特优势，积极发展汽车配件、汽车模具和装备制造产业。用足用好国家、省扶持新兴产业发展政策，完善我市激励政策措施，大力发展半导体照明、新材料和绿色能源三大新兴产业。紧紧抓住我市便于南融北拓的桥头堡区位和紧邻广州空港产业区的优势，规划建设现代物流产业集聚区。争取设立保税物流中心，做大商贸、现代物流仓储产业，加快发展电子商务、金融、保险等生产性服务业。

优化产业发展平台。根据园区资源、区位条件和现有产业基础，进一步完善园区规划，科学确定园区的产业定位，逐步培育符合园区实际的特色主导产业。创新园区开发建设模式，按照打造产业新城的目标，引进有实力的园区配套企业，高标准、整片推进园区生产生活配套设施建设，增强园区对优质企业的承载力和吸引力。积极鼓励和支持有实力的民营企业，整合土地资本、金融资本和产业资本，开发建设科技产业园。

增强产业创新驱动。整合政府扶持资金，定向扶持一批具有核心自主知识产权的企业，加快培育一批创新型企业。深入推进产学研合作，支持企业、工业园区、特色产业基地和专业镇与高校、科研机构合作，共建研发中心和创新服务中心。加强知识产权保护工作，鼓励企业和行业建立国际标准跟踪机制，积极参与行业标准和国家标准的制定与修订。

提高产业引进质量。确立从容选商的理念，提高产业准入门槛，综合考虑项目的环境影响、能耗水平、投资强度、税收贡献，并按项目质量供应产业资源，做到项目跟着规划走，用地指标跟着项目走。充分利用省职教基地落户清远的效应，吸引对技工人才需求大的产业和企业投资设厂。进一步完善招商引资政策，按照集群招商的思路，积极主动对接龙头企业，以龙头企业带动上下游企业，做到引进一批企业，形成新的优势产业。

（二）提升城市化水平

按照“一心两核”的区域城市空间布局，集中打造中心主城区和英德、连州两个次中心城市，带动其他中小城镇的发展，增强城市服务区域和产业发展的功能。

打造绿色湖城。主城区的山水条件得天独厚，完全有条件建成全省最美的城市之一。把主城区打造成绿色湖城，是富有灵气的构想，也是主城区的特色所在。要确立人本城市的理念，将市民的安全、便利、舒适作为城市建设最大的价值取向。不比拼城市规模，不追求高楼大厦，着重做优城市环境，让山的绿色和水的蓝色渗透到城市，营造出半城山色半城湖的景观，真正打造一座生态宜居城市，在提升市民生活质量的同时，吸引更多的人才到清远就业创业，为发展高端产业创造条件。用五年时间，完成湖城主体水系建设、城市景观中轴线和一批重点工程建设，奠定湖城格局。加快“三旧”改造力度，建设一批城市综合体，加快发展商贸业，完善城市服务功能。充分发挥主城区紧临大广佛都市圈的优势，主动对接珠三角，加快融入大广佛。主动与广州、佛山建立城市战略合作关系，承接广佛都市圈外移的功能，推进连接广佛都市圈的重大交通项目建设，实现与广佛都市圈的便捷联系，推动广佛清一体化发展。

推进英德、连州次中心城市建设。拉大英德、连州的城市框架，优化城市空间布局，完善城市功能，打造成中、北部地区的区域中心城市，与主城区形成主副功能互补格局。加快推进出省通道，以及连佛高速等连接中心城区、周边地区的交通网络建设，争取柳韶铁路建设纳入国家规划。完善城市主干路网，营造富有特色的城市环境，提升城市综合服务水平，增强吸纳人口和集聚产业能力。引导英德、连州按照主体功能区规划，立足本地资源优势，正确处理开发建设与生态环境保护的关系，以园区为载体，大力培育特色主导产业，将英德建设成为珠三角产业转移的重要承接地，将连州建设成为粤、湘、桂结合部具有较强辐射力的区域中心城市。

完善县城和中心城镇功能。深化县级城市总体规划，做强县城和中心镇，适度扩大城市规模，完善基础设施、生活配套和社会管理功能，积极引导产业向中心镇集聚，促进农村人口向中心镇集居。用五年时间全面完成镇村的规划编制，突出抓好镇村布局、新村建设和旧村改造规划，实现城乡规划无缝对接，全市域覆盖。打造一批名镇名村，建设独具岭南特色、生态宜居的美丽乡村和美好家园。

（三）推进区域协调发展

城乡和区域发展不平衡，是我市实现新跨越的“短板”，也是建设幸福清远的难点所在。必须统筹区域发展，不断缩小南北部经济社会发展差距，努力建设全省区域协调发展示范区。

推进主体功能区建设。严格按照主体功能区规划来推进开发建设。建立生态补偿机制，为生态发展区和禁止开发区更多地专注于生态保护提供支持。通过调整完善市级财政统筹、考核评价和人口转移等机制，强化分类管理，推进差异化发展，确保有限的工业发展资源更多集中配置到南部适合发展的区域和英德、连州两个新的增长极，逐步构建优势突出、特色鲜明的主体功能区。

加快市域重大基础设施建设。加快形成“五纵三横”高速公路网，实现县县通高速目标。加快推进广清城际轻轨、北江航道改造等重大项目建设，积极推进国、省道区域干线公路和县、乡道的升级改造。加快完善电网，全面推进燃气工程建设，满足快速增长的能源需求。大力发展民生水利，提升防洪除涝减灾设施保障水平，完善城乡居民供水安全保障体系。加快生态景观林带建设。

提升欠发达地区公共配套水平。进一步加大财政投入，引导社会资金参与，优先推进北部山区、民族地区基础设施和公共服务建设，不断优化欠发达地区发展环境。加快农村公路养护体制改革，积极实施公路安保工程，完成3000公里以上的农村公路改造。加快北部山区和民族地区农田水利、灌排工程和治洪治涝工程建设，争取“整市推进村村通自来水”工程纳入省计划。大力扶持北部山区和民族地区教育、文化、卫生等事业的发展，提高基本公共服务均等化水平。

（四）增强区域软实力

围绕提升文化竞争力、增强聚才育才能力、加快体制机制创新，全面增强区域的软实力。

提升文化竞争力。着眼于人的全面发展，培育以社会主义核心价值体系为灵魂、以岭南优秀文化传统为底蕴、以现代文明素质为特征的时代精神，推动全市上下的观念更新，形成有利于清远新跨越的主流价值取向。全面实施文化强市规划纲要，加快城乡文化基础设施建设，健全城乡公共文化服务网络，实施文化惠民工程，力争到2015年，基本完成广播电视节目数字电视整体转换，全部乡镇（街道）建有综合文化站，全部行政村（社区）建有文化室。丰富传统文化内涵，积极开展群众性文化活动，打造具有清远特色的文化品牌。深化文化体制改革，扶持文化产业发展，推动文化与旅游、体育等相关产业的融合发展，延伸文化产业链条。加强文化遗产保护和利用，鼓励文艺精品创作。努力争创全国文明城市。

增强聚才育才能力。确立“不求所有，但求所用”的理念，完善人才引进的政策体系，加强与高等院校、科研机构和人才租赁公司的沟通合作，采取任职、兼职、顾问、合作开发和创建、承包租赁实体等方式，多渠道引才引智。完善人才服务体系，大力实施“十百千万”人才行动计划，着力引进专业技术人才、经营管理人才、技能人才、复合型人才和重点学科或学术带头人。加快推进职教新城建设，抓好各类专业技能培训，尽快培养一批适应产业转型升级的急需人才。制定人才继续教育规划，提高人才素质。实施灵活多样的人才柔性流动政策，稳定现有人才，鼓励和吸引人才到贫困山区、民族地区工作。

加快体制机制创新。推进现有社会组织实行“去行政化”和“去垄断化”改革，建立社会组织孵化基地，改进社会组织登记办法，放宽准入限制，降低登记门槛，大力培育和扶持社会组织发展。促进社会组织自律管理、自我规范。加大政府购买服务的力度，积极推进政府职能向社会组织转移。理顺市县两级管理权限，加大向县（市、区）放权的力度，凡是有利于县（市、区）经济社会发展的审批和公共服务事项，尽可能下放给县（市、区）管理，让有条件的县（市、区）有足够的发展自主权，进一步做大做强县域经济。积极下放城市管理和社会服务管理等职能事项，改变市级政府包揽过多的状况。稳步推进山区县农村综合改革，扩大镇级政府行政事务管理和处置权限，落实村民自治，促进管理重心下移。加快组建农村商业银行和村镇银行等新型农村金融机构，构建多层次的融资体系。整合相关国有资产，建立政府投融资平台，以金融创新筹集资金，推动城市建设与产业发展。

（五）提高民生建设水平

加快推进以改善民生为重点的社会建设，创新保障和改善民生的思路举措，把尊重民意、吸纳民智、凝聚民心贯穿于民生工程全过程。

完善公共民生服务。以创建省教育强市为抓手，全力推进城乡义务教育均衡发展，保障困难家庭子女、残疾少年、非户籍常住人口子女平等享受义务教育的权利。普及学前三年教育，提升高中教育、技工教育和高中职教育发展水平，力争清远高职院创建成为省示范性高职院校。尽快增加公办优质学位，解决中心市区优质小学学位紧缺问题，办好让人民满意的教育。整合卫生资源，推进医疗卫生资源重点向农村和社区倾斜，健全农村和社区卫生服务网络。加强公共体育设施建设，广泛开展全民健身活动。完善公共交通线路、枢纽站场的规划建设，方便群众的出行。

加强基本民生保障。建立健全以中低收入住房困难家庭为保障对象，以公租房为供给主体的新型住房保障制度，多形式建设保障性住房，力争到“十二五”期末，基本解决我市人均住房面积小于13平方米、家庭人均月可支配收入低于全市低保收入线上浮200%的人员住房保障问题。深化医疗保障制度改革，积极探索商业保险机构参与医保管理服务的新路子。加快建立统筹城乡的基本医疗保障制度，推进新农保和城镇居民养老保险“三年全覆盖”工作。完善低收入群体临时价格补贴与价格上涨联动机制，建立综合性社会救助体系，多渠道建设养老服务机构。进一步扩大低保、五保覆盖面，提高保障水平，确保困难群众温饱无忧、体面尊严地生活。巩固“双到”扶贫开发成果，建立和完善贫困地区群众脱贫致富的长效机制；完成高寒山区移民搬迁和农村危房改造任务。

解决热点民生问题。实施更加积极的就业政策，切实抓好困难群体的就业援助，推进职业技能培训扩量提质，提高统筹城乡就业服务水平。建立健全基层劳动争议调解组织，加强劳动监察柔性执法，维护劳资双方合法权益。切实抓好安全生产，防止重特大安全事故的发生。完善治安防控体系建设，打击和惩治各类犯罪活动，着力解决影响群众安全的突出治安问题。把稳定物价放在更加突出的位置，确保粮、油、肉、蛋、菜等生活必需品市场平稳运行。健全食品药品安全保障和监管长效机制及安全预警应急机制，严把食品药品生产质量关，狠抓市场监管，确保产品质量和食品药品安全。

（六）建设廉洁高效务实政府

依法行政，激励创新，优化服务，推动政府职能真正向创造良好发展环境、全面加强社会管理、提供优质公共服务、维护社会公平正义转变。

建设廉洁政府。强化廉政风险防范管理工作，重点加强对权力部门和权力岗位的监督，针对土地出让、工程招投标、政府采购、项目审批、资金安排等政府工作中容易滋生腐败的环节，加快制度的建设和完善。深化财政管理体制改革，严格实行收支两条线，规范专项资金和各类基金的使用。加强舆论监督，健全行政听证、社会公示、专家咨询、决策评估等制度。强化审计监督、执法监察、廉政监察和效能监察，完善对重点项目、重大活动、重要资金使用的专项督查制度，坚决查处违法违纪行为，坚决纠正损害群众利益的不正之风。

建设高效政府。按照公开、透明、便民、高效的要求，深化行政审批制度改革，推行协同办公和联合审批，压减审批时限。总结清城区大部门制改革试点经验，积极推进职能有机统一的大部门制改革，加快形成有利于统筹发展、提升效能的行政管理体制。加强政府信息化建设，加快建成集政务公开、行政审批、信息发布、政府采购、公共资源交易、行政投诉受理、电子监察于一体的综合政务服务平台。

建设务实政府。崇尚实干，全面推行“问题”管理工作模式，以解决存在问题为抓手，有效提升政府执行力。强化对企业的服务，建立政企定期沟通协调制度，及时解决企业发展过程中需要政府解决的问题。高度重视社情民意，强化督促指导和现场办公，及时解决社会关注的热点难点问题。减少文山会海，集中精力抓发展、抓落实，确保工作定一件、干一件、成一件。

三、2012年的工作安排

五年宏图，重在开局。今年是实现清远新跨越的起步之年，也是落实桥头堡发展战略的破题之年。展望今年的内外形势，开局之年的工作将面临较大的挑战。从国际看，世界经济下行风险明显加大，外需市场疲软，出口型企业生产经营的难度加大；从国内看，国家宏观调控效应进一步显现，房地产等主要依靠银行资金运作的行业和企业将面临更大压力。从我市的情况看，传统的产业体系、发展方式已经无法为经济增长提供足够的动力，我市经济在原有发展方式上的高速增长已近极限，经济减速下行与社会矛盾增多的趋势令人警惕。特别是，全市工业投资已经连续多年明显回落，新增产能、税源不多，全年的经济增长缺乏足够的支撑。

统筹考虑上述因素，今年政府工作的总体要求是：深入贯彻党的十七届六中全会、中央经济工作会议、省委十届十一次全会和市第六次党代会精神，把握稳中求进的工作总基调，以落实桥头堡发展战略、推动转型升级为抓手，加大重点建设项目的投入，保持全市固定资产适度的投资规模；加大对实体经济的帮扶，通过稳定企业的生产经营来保持全年的经济增长；加大推进产业提升的力度，扎实推动发展方式的转变；加大民生建设的力度，让群众充分分享经济发展的成果；最大限度地减少不和谐、不稳定因素，保持社会大局的和谐稳定。

按照上述总体要求，建议今年经济社会发展的主要预期目标和约束指标是：生产总值增长12.5%，人均生产总值力争实现同步增长；固定资产投资增长15%；地方财政一般预算收入增长13%；实际利用外资增长3%，外贸出口总额增长6%；社会消费品零售总额增长15%；城镇居民可支配收入增长10.5%以上，农村居民人均纯收入增长11%以上，城镇居民登记失业率控制在3.5%以内，居民消费价格总水平上涨幅度控制在4%以内；二氧化硫、化学需氧量排放、万元生产总值能耗下降率完成省定任务。

这里需要特别说明的是，与往年相比，我们适当调低了今年的经济增长预期，除了基于国内外形势的判断外，要重要的是希望通过主动放缓增长速度，换取结构调整的空间，引导全市经济转向有质量、高效益、可持续的增长，为今后几年实现又好又快的发展创造条件。

围绕实现今年的预期目标，建议重点安排十项工作：

（一）提升园区发展水平

加快园区基础设施配套。在完善园区规划的基础上，引进有实力的企业，加快经济开发区、华侨工业园、民族工业园的主干道路、市政设施及基本的生活配套设施建设，力争明年上半年基本完成。

理顺市级园区管理体制。理顺经济开发区、华侨工业园、民族工业园与所在地政府、市直部门的关系，最大限度地对园区下放管理权限。用制度确保顺德清远（英德）经济合作区由顺德区主导，把涉及园区的政府审批权限，全部转入园区管委会，建立园区管委会与省政府的直接请批关系。加大园区申报整合力度，争取民族工业园创建成为省级产业转移园。

加大园区招商力度。清理闲置工业用地，研究制定闲置土地的统筹招商办法，提高园区的项目准入门槛。在开发区规划建设汽车配件制造园区和光电产业园区，做好与珠三角汽车配件企业和半导体照明企业的对接，力争年内有部分企业入园。

推进园区科技进步。力争培育省级以上创新型企业3家。支持高新区建设区域创新中心。加大县域科技创新能力的培育。推动一批国家级、省市级工程技术研发中心、技术创新平台、科技孵化器和创新服务中心落户园区。

整合提升传统产业园。继续引导金属拆解企业进入石角园区，加快园区的公共配套建设。争取海关、检验检疫等部门的支持，在石角建立虚拟的废品废料进口监管园区。落实重金属污染防治规划，抓好重点行业和重点企业污染整治。清理整治市中心区域废旧物资拆解场。鼓励、扶持企业在余热余压利用、节约能源和“三废”综合利用等方面开展技术改造和创新。建设陶瓷企业总部基地，引导陶瓷企业逐步把营销、工艺设计等总部要素转入清远，延伸产业链，增加陶瓷产业的附加值。

加快服务产业发展。完善旅游业发展规划和清远旅游服务中心，理顺飞霞风景区管理体制，加快组建运作北江旅游开发投资集团。推动已引进的一批规模较大的旅游投资项目加快建设。大力开拓珠三角、沿海和境外高端旅游客源市场。抓好清远中汽南方国际汽车城、义乌商贸城二期、华南装饰城、粤北农产品批发中心的建设。解决好预备上市企业的土地产权等历史遗留问题，实现清远上市企业零的突破。

（二）实施湖城建设工程

完成湖城规划修编。上半年完成《清远市城市总体规划》、《清远市中心城区城市设计》以及《清远市湖城水系与景观规划》等专项规划的修编工作，年内完成重点地区的控制性详细规划。完善城市规划和重大建设项目公示制度，让市民更加深入地参与湖城的规划。

启动湖城新区的规划建设。成立城市开发投资公司,着手湖城的框架工程建设。对北江四桥及新城市中轴线，新城中心片区、大燕湖片区等重点区域进行规划设计招标。启动银盏温泉特色小镇示范点的规划建设。启动南山岭等一批公园、绿地的规划建设。

推进重点市政工程建设。继续推进城西大道二期、富强路二期、北江四路、新区二期、飞来湖周边道路等在建道路的建设，确保年内完成。按照堤路结合的方式，加快北江东路至武广火车站道路改造提升。启动民安路、凤翔南路延伸段、武广客运站站前东路、清晖北路、凤鸣西路等一批骨干道路桥梁的建设，打通汇祥路、松鹤街两条新旧区连接线，加快新旧城区功能对接。增加市区的休闲健身设施。成立市水务集团，着手进行市区第二、第三水厂及文洞水库备用水源的规划设计与建设，加快横荷、东城、乐排河污水处理厂及配套管网建设。推进大燕湖、大燕河水利枢纽和青榄海古河道疏浚整治工程，完成清远水利枢纽工程建设，制定办法对库区水质进行保护。

（三）加快市域基础设施建设

加快交通工程建设。全力配合广乐、二广高速和广清高速扩建工程建设，解决好征地拆迁的遗留问题。着手佛清从高速北段的征地拆迁工作，力争下半年动工。积极做好汕湛、汕昆高速清远段和连佛高速的前期工作，为项目尽早建设创造条件。完成清连二级公路大修收尾工程和清三、清佛公路大修工程。力争银英公路扩建工程上半年完成。推动106国道佛冈段、连怡公路、英坑公路年内动工大修。积极推动广清城际轨道早日动工建设，配合完成武广专线英德客运站建设。推进北江千吨级航道建设前期工作，恢复清远港功能，加快提升通航能力。

加快水利工程建设。出台并启动民生水利五项工作方案，完成3宗中小河流治理工程、9宗重点小（二）型病险水库除险加固工程、14宗农村重点小型机电排灌工程等水利工程建设。基本完成枫树坪中型水库和茅舍岭水闸除险加固工程。

加快供配电及信息工程建设。加快500千伏库湾输变电站220千伏配套工程、220千伏石潭输变电以及110千伏清远清新江口、英德富强等一批输变电工程建设，逐步优化和完善110千伏电网。启动西气东输工程清远燃气利用项目，完成1公里输气管道和27公里次高压管道的建设，确保10月前投产通气。推进“无线城市、数字企业”信息化应用工程建设，加快应急指挥平台、医疗数据系统、电子图书馆等14个信息化项目的建设。

（四）落实主体功能区规划

制定主体功能区分区规划。按照总体规划的要求，加快制订各县（市、区）的主体功能区分区规划，确保上半年完成，推动主体功能区规划的全面实施。

建立生态补偿机制。探索建立下游地区对上游地区、开发地区对生态地区的利益补偿机制，重点支持生态发展区和禁止开发区的基础设施、生态环境保护和产业发展。完善生态公益林补偿制度，积极探讨水权交易和排污权交易机制。

建立财政转移支付机制。市级财政在预算内安排专项资金，并对重点开发区新增税收实行市县分成，由市统筹用于加大对生态发展区和禁止开发区的财政转移支付，保障这部分地区的公共服务水平和基本支出水平。

完善考核评价机制。制订新的市对县（市、区）工作考核办法，针对各县（市、区）不同的发展定位和工作重点，制定不同的考核指标，实行差异化考核，引导各地真正按主体功能区规划，实行差异化发展，为加快形成主体功能区打好基础。

建立人口转移机制。研究制定专门政策，完善土地流转办法，鼓励引导生态发展区、禁止开发区的劳动力和人口向区域中心城镇转移，支持中心城镇的产业发展，并降低这部分地区土地供养人口的压力，更好地实现区域发展的均衡。

（五）扎实开展扶贫开发

巩固扶贫开发“双到”工作成果。建立互助金示范村200个、脱贫示范村100个，确保50%的贫困村建立起村级脱贫长效机制。采取“订单、定向、储备”的培训模式，继续实施“一户一技能”培训计划，对符合条件的贫困户劳动力开展免费职业技术培训，确保每户至少输出一个劳动力。进一步完善贫困户与龙头企业的互助联结机制，以产业化带动贫困农民全面脱贫。

加快“两不具备”移民搬迁。确保完成搬迁4130户18805人，为“五年任务三年完成”打下坚实基础。加快推进农村贫困户危房改造工作，建设和完善33个幸福安居示范村，完成302个省级贫困村村庄规划及1.74万户的危房改造任务。

扶持民族地区发展。认真梳理国家和省扶持民族地区发展的政策，加强与省相关部门的沟通对接，促进民族政策的全面落实。支持连山县德建水库年内开工建设，推进连山永福联围、阳山秤架河中小河流治理工程年内建成。推进华润风力发电项目落户连山。支持民族地区特色农产品、民族医药健康产业基地建设，加快培育优势产业。积极推动连山、连南两个民族自治县纳入国家级生态保护区。认真做好民族地区非物质文化的传承。加快广东瑶族博物馆建设，力争年内建成开馆。

（六）推进农业农村建设

实施农业“两品工程”。扶持沙糖桔、优质蔬菜、蚕桑等特色产业发展，加快清远现代农业示范区建设，新建一批具有一定规模的有机农产品生产基地、现代农业园区。加大农业技术推广应用的投入和水果病害的防控。积极争取省有关部门的支持配合，办好省、市农民专业合作组织试点，引导合作组织向规范化方向发展。引导龙头企业强化品质意识和品牌意识，力争年内打造绿色食品认证10个、有机食品认证8个、省名牌产品2个。督促大型养殖企业加强环保治理。

完善农田水利设施。继续推进现代标准化农田建设，建设基本农田示范区2个，共1万亩；完成农业综合开发5.17万亩，农田水利基本建设9千亩，基本农田整治5千亩；改造中低产田1千亩。

加快完善农村基础设施。继续推进农村电网改造，不断满足快速增长的用电需求。全力推进村村通自来水工程建设，确保农村饮水安全。完成500公里通自然村公路路面硬化工程。深入开展“农村公路建设质量年”活动，继续实施公路安保、危桥改造等工程，完成12座危桥改造任务。

积极推进名镇名村建设。加快编制市、县（市、区）名镇名村及示范村发展建设规划，挖掘、保护改造一批有传统文化特色的村镇，力争每个县（市、区）打造2条以上的名村和示范村。狠抓农村环境卫生整治，开展农村污水处理建设试点工作。推进佛冈县龙南片区“广东省社会主义新农村建设实验区”建设，确保年内有实质性突破。加快“四有示范村”建设，力争示范村农民人均收入增幅比全市农民人均收入增幅高3个百分点。

扎实推进山区县综合改革。制定农村综合改革配套政策，抓好英德、连州、清新的农村综合改革试点工作，重点抓好乡镇机构改革和村民自治的试点。

（七）积极引进与培育人才

细化人才政策。实施人才智力支持贫困山区、民族地区发展行动计划，采取提高待遇、定期轮换等措施，稳定贫困山区和民族地区现有人才，并吸引一批各领域的专家、技术骨干、大学生志愿者到贫困山区和民族地区工作。对专业技术拔尖人才发放技术津贴。设立“优秀人才创新奖”，对有突出贡献的优秀人才给予表彰奖励。鼓励企业对经营管理人员和技能人员实行重奖。

搭建引才引智平台。将珠三角成熟的人才市场引入清远，提升现有人才市场服务水平，推进人才市场体系建设，搭建人才引进平台。聘请各领域的专家学者，担任政府高级顾问，提升政府科学决策水平。

加快推进省职业教育基地建设。尽快组织职教城的规划设计招标，力争明年上半年完成。加快职教城的征地拆迁、村民安置等前期工作，制定开发建设计划，确保下半年基础配套设施开工建设。加强与意向院校的对接，选择一批有实力、符合职教城发展要求的院校进驻。

（八）大力发展社会事业

优化发展教育事业。办好乡镇公办中心幼儿园，制订幼儿园民办公助办法，对符合条件的民办幼儿园给予办学资助。理顺市区办学体制，调整市区学校布局，启动市新一中、新城小学建设，加快清远高职院创建省示范性高职院校步伐。进一步调研论证县以下教育资源布局，方便农村学生就近读书。加强校车交通安全管理，推进新一轮“校安工程”，保障学生安全。制定教师队伍建设“十二五”实施意见。

加快发展文化体育事业。继续办好广场文化节、艺术活动月。深入实施广播电视户户通、文化信息资源共享、农村电影放映、三馆一站免费开放、农家书屋等文化惠民工程。深入挖掘、保护传统特色文化资源，对全市有价值的传统物质文化和非物质文化资源开展一次普查，并制订保护和开发规划。深入开展全民健身运动，建设19个乡镇农民体育健身场所，支持恒大皇马万人足球学校建设。

着力发展卫生事业。进一步整合医疗资源，将市人民医院打造成为区域性综合医院，着重于全市疑难重症的治疗及教学、科研以及人才的培养；将市妇幼保健院建设成具有一定水平的专科医院；突出中医特色，提升市中医院综合服务水平。创造条件引入高层次医疗资源，提高我市医疗服务水平。健全社区卫生服务系统，积极发展社区门诊。认真贯彻落实省《关于加强计生工作促进人口长期均衡发展的决定》，完善和创新人口服务管理机制，按甲级标准重建市计划生育服务中心，完成人口计生信息系统的改造升级。

扩大公共交通覆盖面。制定《清远市公共交通优先发展意见》，调整优化公交网线，推进城乡公交一体化。实施城市公交惠民工程，做好市民卡与省一卡通的对接工作，推动市民卡在一票制公交线路上全面应用。转变农村班线经营机制，逐步解决个体承包挂靠经营问题。创新农村客运站（亭）养护机制，提高农村客运能力。

深化社会民生领域改革。积极稳妥推进公立医院改革。进一步扩大基本药物制度覆盖范围，规范基本药物的采购和使用。推进医疗卫生系统信息化建设，实现全市卫生信息资源共享。推进城镇职工基本社会保险、新农保和城镇居民养老保险扩面征缴工作，稳步扩大基本医疗保障覆盖面。建立完善市县两级医疗救助制度，建立健全社会福利服务体系，提高五保保障水平，发展居家养老服务。制定住房保障制度改革实施办法。切实抓好食品药品监管和价格监督工作，狠抓安全生产、信访维稳和社会治安综合治理，化解社会矛盾，提高群众安全感。积极实施《妇女儿童发展规划》。建立健全殡葬基本服务保障制度，提高殡葬服务水平。

（九）认真办好十件民生实事

在完成2011年政府承诺办好的十件民生实事的基础上，经过论证和广泛征求市民意见，今年市县两级财政将投入资金5亿元，集中精力办好以下十件民生实事：一是提高养老服务水平。改善老年人生活，建立全市80岁以上高龄老人津贴制度。二是继续加大扶贫力度，改善农村贫困群众的居住条件。完成我市农村低收入住房困难户危房改造8007户任务，以及不具备生产生活条件贫困村庄搬迁安置4130户、18805人任务。三是抓好市中心区慢行系统建设。完成飞来湖周边绿道、北江市区段南岸绿道建设，修复白庙至飞来寺2.9公里的旅游古栈道。四是继续抓好保障性住房建设。解决符合住房保障条件的2505户家庭住房困难问题，完善保障性住房准入和退出机制。五是改建好群众艺术馆及文化广场。普及广场文化活动，提升活动水平。六是改造提升肉菜市场。在市区和各县县城选取10个肉菜市场进行升级改造，打造样板市场，并制定政策逐步推动其他肉菜市场进行提升改造。七是着力解决“入园难”问题。在全市新建、改造30所规范化公办幼儿园，增加学位，提高质量。八是推进欠发达地区的农田水利设施建设。完成14宗农田水利小型灌区改造工程任务，改善农业发展条件。九是提升基层医疗服务水平。全市所有中心镇卫生院和村级卫生站建设实现达标。十是健全市区公共交通体系。增加（延伸）公交线路，提高营运能力，并抓好市区5条主干道交通安全设施建设，促进市区交通秩序明显好转。

（十）切实提升政府执行力

加强行政服务中心建设。设立网上审批大厅，实现全市审批一网通。转变审批方式，改串联审批为并联审批，提升审批效率。完善电子监察系统，对重点项目审批和行政执法进行全方位监督。

加强机关作风建设和公务员培训。进一步推行服务承诺制、首问负责制、限时办结制等制度，提高执行力和抓落实的能力。建立机关作风与效能建设社会评议评价体系，开通机关作风与效能建设网络评议平台。制订《清远市机关治理庸懒散问责暂行办法》，加大责任追究力度，以“硬措施”治理“软环境”。加强公务员职业能力培训和新兴产业知识学习，全年培训1.78万人。优化公务员队伍知识结构，提高推动科学发展的能力。

推进行政管理改革。进一步深化市级机构改革，按照大部门制的要求，调整撤并部门设置。新设立市社会工作委员会，加大社会建设、管理、服务的力度。制订向县级放权的实施办法，进一步明确市、城区在城市管理监察、绿化、卫生、交通管理等领域的职责。建立统一规范、公正透明的政府购买社会服务制度，出台市政府向社会组织依法转移政府职能和购买服务目录。改革财政预算管理办法，严格实行收支两条线，改革专项资金和各类基金的管理，加强财政的统筹能力。压缩一般性支出，降低行政成本。严格控制非生产性、非民生类项目的投入，真正将资金集中用于重点民生项目和有利于形成新增长点的领域。

各位代表，国防动员和国防后备力量建设，始终是政府的一项重要工作。要以继续争创省双拥模范城市为载体，扎实开展国防教育，认真做好国防动员，深入开展双拥共建活动，巩固和发展军政军民团结。

各位代表，过去五年，我们创造了非凡的业绩；未来五年，我们将续写新的辉煌。让我们在市委的坚强领导下，用新跨越的共同愿景凝心聚力，以更多的智慧和心力，以更务实的工作作风，在历届班子“一棒接一棒”打下的良好基础之上，继往开来，奋发进取，全面落实桥头堡发展战略，浓墨重彩地抒写推进清远经济社会发展新跨越的崭新篇章！
